# Investigation on heat transfer mechanism simulation and structure optimization design of hydraulic turbine bearing semi-ring cooler

**DOI:** 10.1016/j.heliyon.2025.e42328

**Published:** 2025-01-30

**Authors:** Yongfei Wang, Yinhui Cai, Jian Zhang, Zhenyu Chen, Chenhao Li, Weipeng Sun

**Affiliations:** a CHN Energy Dadu River Repair & Installation Co., Ltd., Leshan 614900, China; b State Key Laboratory of Eco-hydraulics in Northwest Arid Region, Xi'an University of Technology, Xi'an 710048, China

**Keywords:** Bearing cooler, Heat transfer, Structure optimization, Spiral twisted flat tube, Numerical simulation

## Abstract

Bearing is an important support to carry radial force and axial force, when hydro generator set is running, a large amount of heat produced by the rotor shaft and bearing touching will yield oil mist and jeopardize equipment health. The cooler can take away the heat in time, therefore, improving the cooler's efficiency is of great significance. In this paper, the internal structure of a turbine water guide-bearing semi-ring cooler at a hydro plant is optimized and numerical calculation is carried out to study heat transfer performance under three operating conditions. The results show that, the proposed spiral twisted flat tube cooler effectively reduces the risk of heat exchange tube blockage and significantly improves cooler heat exchange efficiency. Compared to prototype cooler, heat transfer power for the optimized cooler with spiral twisted flat tube increases by 43.8%, 62.2%, and 73.8%, respectively. Optimizing spacer plate positions at the cooler's inlet and outlet further enhances heat transfer power by 47.2%, 52.4%, and 56.4%, respectively. The pressure, velocity, and other parameters of cooler are also improved after both types of optimization compared to the prototype. The findings provide a reference for improving the thermal performance of guide bearings.

## Highlights

• The heat transfer equation for the cooler is presented.
• Operating principle of the radiator is explained using numerical simulation.
• The application of spiral flat pipe adds the heat transfer coefficient and area.
• Optimization of the bulkhead position results in increased heat transfer coefficient.
• Both optimized solutions provide significant improvements in cooler performance.

## 1 Introduction

As an important energy generation facility, hydroelectric power station can convert water energy into electricity. When the unit works, the generator set produces numbers of heat, which needs to be effectively dissipated by the cooler to ensure the equipment's running and improve power generation efficiency [1], [2], [3]. Most coolers in hydro station are shell-and-tube coolers, which play a major role in coolers for simple structure, low manufacturing cost, and wide adaptability [4], [5], [6]. The heat transfer enhancement increases heat exchange as much as possible per unit time and unit area under the same power consumption. And the main enhanced heat transfer tubes include bellow [7], transverse tube [8], spiral flat tube [9], [10], retractable tube [11], and finned tube [12].

On the one hand, for hydro station affected by the outdoor high-temperature weather, the condensation temperature of water turbine unit motor, using volute intake or air convection cooling, will rise with the increase of outdoor dry bulb temperature. The output of hydraulic turbine unit will be blocked, which not only weakens the heat dissipation performance of the unit, and also increases the unit's power consumption during operation [13]. Additionally, the rotor shaft rotates at high speed when the hydro generator set is operating, and the transverse and longitudinal loads are transmitted through the guide bearing and thrust bearing. The prolonged touching and contact between the rotor shaft and bearings will cause the bearing tile's temperature to rise, and if the generated heat is not transferred timely, the bearing tile will be easily burned [14].

On the other hand, several practical application studies have been carried out to address the heat dissipation of hydro generator units. Najar et al. [15] installed additional cooling water pipes below the thrust bearing tile, which allowed direct cooling of the high-temperature oil film. And numerical simulation results were employed to illustrate the effectiveness of proposed method. Ettles et al. [16], [17] considered the effect of the thickness for high pressure oil film on heat dissipation, and tested the thrust bearing tile surface made of PTFE material, they finally revealed that, this material mainly affects the thickness of oil film but basically has no effect on the oil film's temperature. Glavatsldh et al. [18], [19] further investigated the bearing tile surface made of various materials and suggested that PTFE material has the most excellent performance of heat dissipation in bearing tile, by comparing the temperature change values of the bearing tile after experiments. Porto et al. [20] increased the number of heat exchangers inside the oil bath of the thrust bearing, and replaced the original shell and tube heat exchanger with a stainless steel plate heat exchanger. This measure significantly reduced the high bearing temperature. Apart from the above, clogging in the radiator piping can also cause inefficient heat dissipation. Frota et al. [21] introduced non-invasive electronic antifouling technology into the heat exchangers of hydro generator sets.

Reviewing the aforementioned studies, during the work of hydro generator set, there will be a large amount of heat generated by continuous contact and collision between the rotating parts and fixed ones. Transferring the heat generated by hydro generator bearings during operation timely plays an important role in unit. Current research has targeted improving the cooler's efficiency, and increasing pipe numbers, unclogging pipes, and improving tank structure have been proposed. However, these methods have the disadvantage of being costly or not being able to fundamentally address the low heat transfer. To solve this problem and create value in the technology of improving heat exchanger efficiency, this paper explores the heat dissipation of hydro generator set under different operating conditions by means of parametric measurements and numerical simulations. Aiming at the existing heat dissipation problems, the heat exchanger is structurally optimized to improve the heat dissipation performance. Which can provide a reference for improving the heat dissipation of hydro generator's bearing cooler in practical engineering.

## 2 Heat transfer model

The water-cooled heat exchanger of hydroelectric generator unit consists of multi-turn pipes, and the flow pattern of water in pipes is turbulent. During turbulence, the water flow satisfies the mass, momentum and energy equations, respectively [22], [23]. Continuity equation (mass conservation equation) is given by

(1) $$\frac{\partial \left(\rho u\right)}{\partial x}+\frac{\partial \left(\rho v\right)}{\partial y}+\frac{\partial \left(\rho w\right)}{\partial z}=0$$

where *ρ* and *u* are the density and velocity vector of flow in Eq. (1), respectively. *u*, *v* and *w* mean the components of velocity vector *u* in *x*, *y*, *z* direction.

The Momentum conservation equation is shown as follows

(2) $$\begin{array}{c} \frac{\partial \left(\rho u\right)}{\partial t}+\frac{\partial \left(\rho uu\right)}{\partial x}+\frac{\partial \left(\rho uv\right)}{\partial y}+\frac{\partial \left(\rho uw\right)}{\partial z}=\frac{\partial }{\partial x}\left(\mu \frac{\partial u}{\partial x}\right)+\frac{\partial }{\partial y}\left(\mu \frac{\partial u}{\partial y}\right)+\frac{\partial }{\partial z}\left(\mu \frac{\partial u}{\partial z}\right)-\frac{\partial p}{\partial x}+S_{u}\\ \frac{\partial \left(\rho v\right)}{\partial t}+\frac{\partial \left(\rho vu\right)}{\partial x}+\frac{\partial \left(\rho vv\right)}{\partial y}+\frac{\partial \left(\rho vw\right)}{\partial z}=\frac{\partial }{\partial x}\left(\mu \frac{\partial v}{\partial x}\right)+\frac{\partial }{\partial y}\left(\mu \frac{\partial v}{\partial y}\right)+\frac{\partial }{\partial z}\left(\mu \frac{\partial v}{\partial z}\right)-\frac{\partial p}{\partial y}+S_{v}\\ \frac{\partial \left(\rho w\right)}{\partial t}+\frac{\partial \left(\rho wu\right)}{\partial x}+\frac{\partial \left(\rho wv\right)}{\partial y}+\frac{\partial \left(\rho ww\right)}{\partial z}=\frac{\partial }{\partial x}\left(\mu \frac{\partial w}{\partial x}\right)+\frac{\partial }{\partial y}\left(\mu \frac{\partial w}{\partial y}\right)+\frac{\partial }{\partial z}\left(\mu \frac{\partial w}{\partial z}\right)-\frac{\partial p}{\partial z}+S_{w} \end{array}$$

where *p* shows the pressure on the fluid micro-element, $S_{u}$, $S_{v}$ and $S_{w}$ are the generalized source terms of momentum conservation equation in Eq. (2). Moreover, energy conservation equation is calculated as

(3) $$\frac{\partial (\rho E)}{\partial t}+\nabla •(\rho uE)=\rho f•u+\nabla •(P•u)+\nabla •(k_{eff}\nabla T)+S_{h}$$

where *f* is the volume force and *t* is the time. *E* and $E_{h}$ refer to the energy and its source term in Eq. (3).

Furthermore, to describe the heat transfer mechanism, the single-phase forced convection heat transfer on the primary side is described using the basic control differential equation as follows

(4) $$\frac{\partial \left(\rho \phi \right)}{\partial t}+\frac{\partial \left(\rho u\phi \right)}{\partial x}+\frac{\partial \left(\rho v\phi \right)}{\partial y}+\frac{\partial \left(\rho w\phi \right)}{\partial z}=\frac{\partial }{\partial x}\left(\Gamma \frac{\partial \phi }{\partial x}\right)+\frac{\partial }{\partial y}\left(\Gamma \frac{\partial \phi }{\partial y}\right)+\frac{\partial }{\partial z}\left(\Gamma \frac{\partial \phi }{\partial z}\right)+S$$

where *ϕ* is the physical variable, *Γ* shows the diffusion coefficient and *S* is the source term in Eq. (4).

Then, the heat transfer differential equation is employed to describe the heat transfer from the primary side to the secondary side through heat exchanger, which is expressed as

(5) $$\frac{\partial \left(\rho_{m}T\right)}{\partial t}=\nabla \cdot \left(\frac{\lambda }{c_{p}}gradT\right)$$

where $\rho_{m}$ is the material density and *T* is the temperature. *λ* and $c_{p}$ represent the thermal conductivity of the material and the specific heat at constant pressure in Eq. (5), respectively.

By analyzing the heat transfer process, the amount of heat transfer per unit time is given by

(6) Q=KSΔt

where *Q* is the total heat transfer of the cooler, *K* and *S* mean the total heat transfer coefficient and area in Eq. (6). Δ*t* is the logarithmic average temperature difference, which is calculated as $\Delta t=(\Delta t_{2}-\Delta t_{1})/In(\Delta t_{2}/\Delta t_{1})$. The heat transfer power *Q* in the inner loop of the cooler is expressed as

(7) $$Q=c_{p}\rho_{m}Q_{V}.(T_{\mathrm{out}}-T_{\mathrm{in}})=c_{p}Q_{M}.\Delta T$$

Where $Q_{v}$ is volumetric flow and $Q_{M}$ is mass flow. $c_{p}$ represent the specific heat capacity of the corresponding medium. Δ*t* is the temperature difference of solution in Eq. (7). When the fluid flow state in the heat exchanger tube and reinforcement tube reaches relative stability, the total heat transfer coefficient can be obtained by

(8) $$K=\frac{Q}{S\Delta t}=\frac{c_{p}Q_{M}.\Delta T}{S\Delta t}$$

In Eq. (8), *K* is the total heat transfer coefficient.

## 3 Physical modeling and numerical calculation

### 3.1 Physical modeling of water-cooled radiator

Thrust bearings and guide bearings are the support equipment for carrying radial and axial forces in hydro generator set. During running, the rotor shaft and bearings continuously collide and friction, which generates a large amount of heat. This heat is transferred to the oil tank installed in bearing housing, and the long-term accumulation of heat will easily vaporize the lubricant to form harmful oil mist. Therefore, a cooling system is required to cool the oil system promptly. The cooler is in direct contact with lubricating oil by being installed inside the oil tank. The heat in lubricating oil is transferred through the cooler's metal tube walls to cooling medium, which flows and therefore continuously separates the heat generated by unit's behavior.

**Figure 1:**
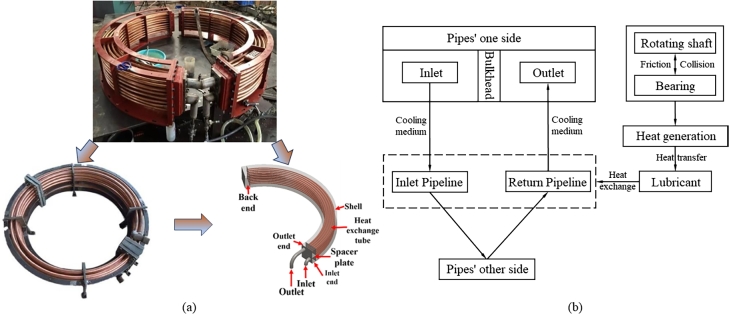
(a) Physical drawing and model construction of a half-ring water-cooled radiator. (b) The components of cooler and their function.

A hydropower plant in China is equipped with seven Francis turbine generator sets of 100 MW capacity. The water-guided bearings of units adopt double-lobe combined cylinder liners, and the lubricating oil is cooled by internal water circulation. To address the specific problems of hydro power plant turbine unit coolers, the heat transfer characteristics are analyzed and optimized based on numerical simulation, where the designed model turbine cooler components are geometrically similar to those of the real machine (aforementioned power plant). Fig. 1(a) shows the physical and modeled drawings of the half-ring cooler (water-conducted bearing oil cooler). In particular, the semicircular cooler consists of multiple turns of piping, which are mounted on a semicircular architecture. The cooling medium flows into the pipelines from an inlet at one end of cooler and returns along the return line after a further semicircular travel, where it exits through an outlet on the same side as inlet. The inlet and outlet parts are separated by a bulkhead to ensure a steady flow of water through the pipeline. As the main heat exchanger, pipeline realizes the heat exchange between the inside flowing medium and outside high temperature lubricant through the pipeline wall, and the low-temperature flowing cooling medium continuously absorbs the heat transferred to pipeline wall by the high-temperature lubricant, which realizes the cooling down of bearings. The components and workflow of cooler are shown in Fig. 1(b).

The cooling for bearings in hydro generator set is based on internal circulation, with coolers arranged in the oil tanks. The volute is used to draw water, and the total cooling water pressure is 0.38-0.4 MPa. The water pressure of the spiral case filter is 0.41-0.42 MPa, and the water pressure in front of the dam is 0.49 MPa. At full load, the water guide cooling tank tile temperature of 2F unit in hydropower plant is 32.7° to 36.9°. To ensure production safety, the alarm temperature and accident temperature are 50° and 55° respectively, and the thrust bearing cooling tank tile temperature is 32.7° to 36.9°. The alarm temperature and accident temperature are 55° and 60°, respectively. The tile temperature of the guide cooling oil tank is 32.7°∼36.9°, and the alarm temperature and accident temperature are 60° and 70° respectively. Table 1 shows the oil cooler's model parameters and actual operating conditions.

**Table 1:** Model parameters and operating conditions for half-ring water-cooled radiator.

Cooler	Pipe size	Materials	Inlet pressure	Outlet pressure
For guide bearing	*ϕ*19 × 1.5 mm	Red copper	0.14~0.17 MPa	0.09~0.1 MPa
For thrust bearing	*ϕ*19 × 1.5 mm	Red copper	0.19 MPa	0.08~0.1 MPa

### 3.2 Condition confirmation for numerical calculation

**Figure 2:**
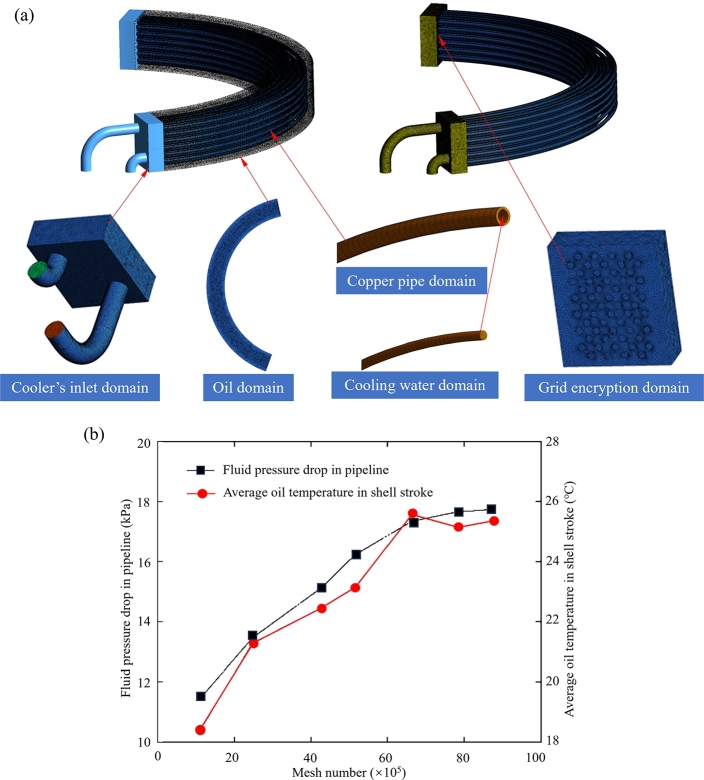
(a) Meshing of semi-annular radiator and (b) mesh-independence test result.

A simplified physical model with geometrical similarity to the actual semi-annular cooler was developed as shown in Fig. 1(a), furthermore, it is shown in Fig. 2(a) that, the described simplified physical model includes a cooler's inlet domain, a high temperature oil domain, a copper pipe domain, a low temperature cooling water domain, and an encrypted region at copper pipe inlet. Since the cooler's inlet domain is relatively complex, the tetrahedral unstructured mesh is used to process this part of the computational domain. Moreover, to ensure the calculation accuracy of heat transfer process for cooler pipe and to effectively control the mesh number, the overall calculation area of other domains is divided into structural meshes. The diameter of a single semi-annular copper tube is 19 mm, the wall thickness is 1.5 mm, and the length is 5 m. Size of the tetrahedral unstructured mesh and the overall mesh are respectively set to 0.1 mm and 0.5 mm. The whole computational domain's meshes are shown in Fig. 2(a). Mesh-independence verification is crucial for numerical simulations, and a suitable number of meshes can ensure the computation efficiency and accuracy. Taking Condition 1 (shown in Table 1) of the prototype heat exchanger model for calculation, since our work divides the cooler into tubes and shells, the variation of fluid pressure drop in pipeline and temperature in shell stroke with mesh number are counted during the grid independence test, as shown in Fig. 2(b). The results show that, when mesh number is larger than 669.2 million, the calculation deviations of pipeline's fluid pressure drop and shell-stroke's average oil temperature are less than 2%. Considering the calculation efficiency and accuracy, the mesh number of 6.692$\times 10^{6}$ is selected for numerical simulation. As the major computational areas, the mesh numbers of the oil domain, the copper pipe wall domain and the water domain are 1.178$\times 10^{6}$, 1.957$\times 10^{6}$ and 3.557$\times 10^{6}$, respectively.

The boundary conditions of the computational domain are further set. Standard wall and no-slip boundary conditions [24] are adopted for the solid wall and the thermally conductive solid is red copper material. The water and oil temperature are set to 298.15 K and 313.15 K, respectively. The inlet and outlet boundary in the computational domain are defined as pressure conditions. While the turbulence model has an obvious effect on the accuracy of flow field calculation, the SST k−ω model, which can accurately predict the separated flow, is employed for numerical calculations.

### 3.3 Confirmation of analytical method

To approach the actual operation state, three pressure values are selected as the boundary conditions of inlet and outlet according to the actual working conditions of bearing cooler. The flow rates in radiator pipe corresponding to the three working conditions are shown in Table 2. Moreover, the total pressure and forward pressure of cooling water under the three working conditions are 0.4 MPa and 0.2 MPa respectively.

**Table 2:** Correspondence between each working condition and the flow rate in pipe.

Working condition	Reverse hydraulic pressure	Mass flow rate	Volume flow rate
Condition 1	0.03 MPa	11.18 kg/s	40.37 m^3^/h
Condition 2	0.13 MPa	21.73 kg/s	78.46 m^3^/h
Condition 3	0.18 MPa	34.65 kg/s	25.11 m^3^/h

**Figure 3:**
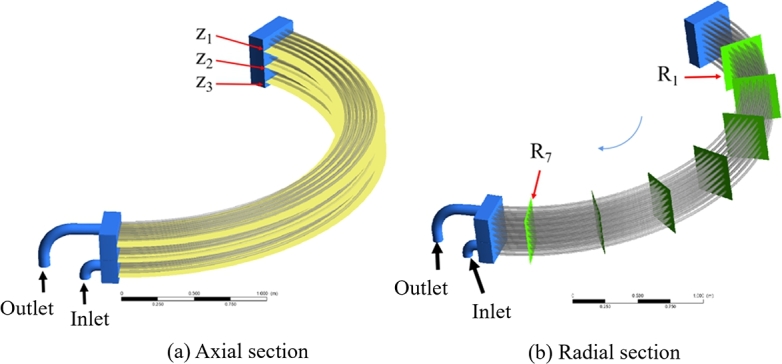
Analytical (a) axial section, (b) radial section.

Typical cross sections shown in Fig. 3 are selected to analyze the water flow and heat transfer patterns in pipes. Fig. 3(a) shows three parallel cross sections equally spaced along the axial direction of turbine unit, designated as $Z_{1}$, $Z_{2}$, and $Z_{3}$, respectively, and Fig. 3(b) displays seven cross sections uniformly distributed along the radial direction, defined as $R_{1}$ to $R_{7}$, respectively.

## 4 Structural optimization and numerical results

Based on the enhanced heat transfer mechanism given in Eq. (6), it is obvious that there are three ways to increase the amount of heat transfer per unit of time, namely, increasing the total heat transfer coefficient *K*, enlarging the heat transfer area *S*, and increasing the average temperature difference between the hot and cold fluids Δ*t*. Furthermore, two improved structures for radiator are proposed based on the enhanced heat transfer mechanism.

### 4.1 Spiral flat pipe structure and bulkhead position optimization

**Figure 4:**
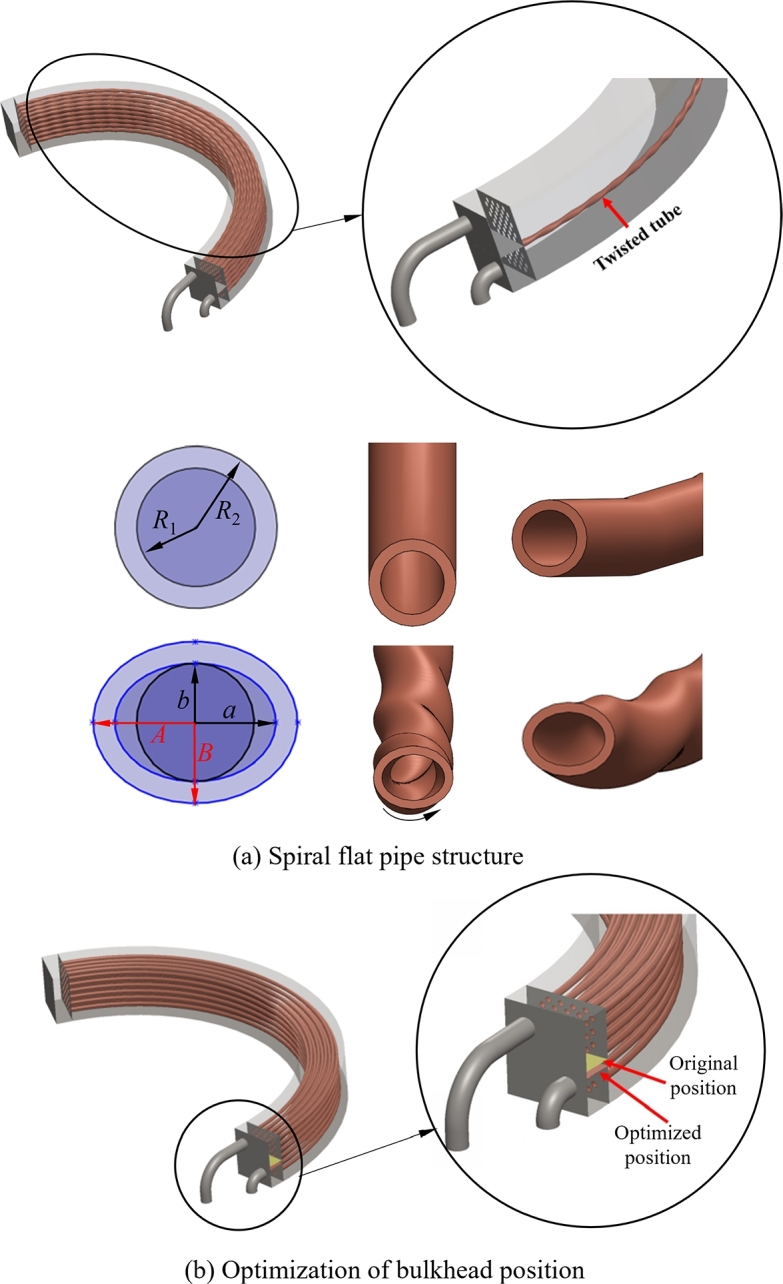
Optimized (a) spiral twisted flat tube structure and (b) bulkhead position.

The traditional cooler with round tube is optimized as a spiral twisted flat tube structure, which is produced by flattening the tube and twisting it into a spiral shape, while the connection between the pipeline and tube sheet is still round, as shown in Fig. 4(a). Based on the tube size parameter of prototype cooler, the inner ring radius $R_{1}$=9.5 mm and the outer ring radius $R_{2}$=13 mm are respectively taken as the short radius and long radius of spiral flat tube's inner ellipse *b* and *a*, and the thickness of pipes remains unchanged with 3.5 mm. The total length of a single heat exchanger tube is 1500 mm, and each spiral length is set to 100 mm, which corresponds to the curvature of 10°. The whole spiral flat tube of the heat exchanger is rotated 15 times, so the total twist angle can be given by 150°. By applying this improved structure to engineering practice, the heat transfer area *S* and heat transfer coefficient *K* can be enlarged, which realizes the enhanced heat transfer by turbulent flow. Additionally, the spiral twisted flat tube is introduced into the design to give full play to the vortex scouring and anti-clogging performance, which realizes the performance characteristics of high efficiency heat transfer and the structure is not easy to be clogged in water-oil bilateral pipes.

Additionally, since the cooling water's flow rate at the end of cooler is slow, the cooling medium accumulates here, and convective heat transfer performance cannot be effectively carried out. To optimize this state, the flow rate and pressure in heat exchanger tube are evenly distributed by changing the bulkhead position, which balances the heat transfer effect between the bottom and top of cooler, thereby a more uniform temperature distribution in pipe is achieved. Adjusting the bulkhead position essentially causes an increase in the heat transfer coefficient *K*. The bulkhead position is optimized as shown in Fig. 4(b), where the bulkhead is positioned towards the inlet.

**Figure 5:**
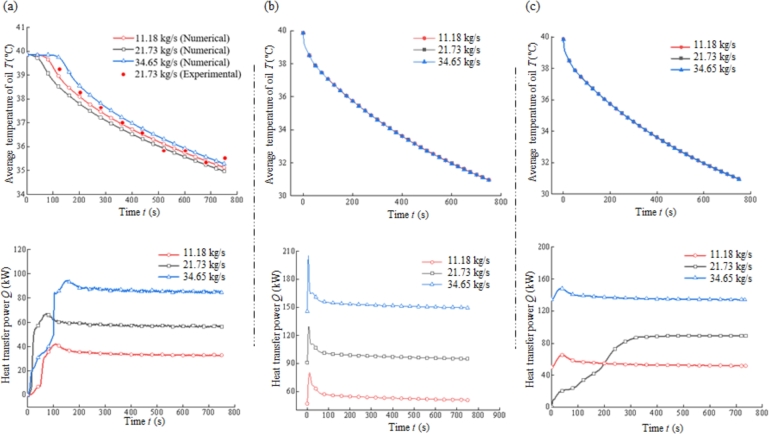
The variation of oil temperature *T* and heat transfer efficiency *Q* with time after numerical simulation for coolers of (a) prototype, (b) spiral flat tube and (c) optimized bulkhead position.

The variation of oil temperature *T* and heat transfer efficiency *Q* with time after numerical simulation are shown in Fig. 5, where Fig. 5(a), (b) and (c) represent the numerical results for coolers of prototype, spiral flat tube and optimized bulkhead position, respectively. Experimental tests were actually carried out using a prototype cooler while the hydroelectric generator unit was running. The technology for accurate measurement of oil temperature in oil tank at the water-guided bearings is already available in hydroelectric power plants, therefore, the prototype was employed at 21.73 kg/s cooling water flow rate for experimental tests to measure oil temperature after cooling as shown in Fig. 5(a). The results show that, when the cooling water flow rate of the prototype is 21.73 kg/s, the trend of oil temperature around water-guide bearing captured by sensor in hydroelectric power plant over time is basically consistent with numerical simulation, and the slight inconsistency between the two is probably caused by the measurement error, and this result also verifies the calculation accuracy. However, considering the necessity to maintain the stable running of the units in hydropower plant, the cooling water flow rate could not be changed arbitrarily, therefore, the experimental validation is only carried out under the working condition of the 21.73 kg/s flow rate in prototype cooler, and the results are sufficient to show that the numerical simulation carried out is accurate.

For the prototype cooler shown in Fig. 5(a), the heat in lubricating oil is taken away by cooler, so that the oil temperature is decreasing under different working conditions. However, there are some differences in the heat transfer speed, in which the average oil temperature decreases the fastest when flow rate is 21.73 kg/s. The heat transfer power under this condition reaches the extreme value around 80 s, which is also the reason for fastest decrease of average oil temperature. The phenomenon that the lowest oil temperature can be reached with a medium flow rate (21.73 kg/s) of cooling water may be explained by the pipe diameter. When the input flow rate is too small, all the water flows directly into pipeline; while when the flow rate is too large, since the pipeline is long enough, a large amount of water can not enter the pipeline in a short time, and some water flows are piled up in the space surrounded by the partition plate and shell, which leads to the efficiency of heat transfer is not ideal in the beginning of heat transfer. After 150 s, the heat transfer power reaches a stable value for different flow rates, and the higher the flow rate, the more heat transfer power. The mass flow rate of 34.65 kg/s, 21.73 kg/s and 11.18 kg/s corresponds to 86.0 kw, 58.7 kw and 35.4 kw respectively.

The results of Fig. 5(b) show that the heat transfer performance in spiral flat tube is approximately the same for different mass flow rates, while the heat transfer power increases with the mass flow rate. The working conditions with mass flow rates of 34.65 kg/s, 21.73 kg/s and 11.18 kg/s corresponded to heat transfer powers of 149.5 kw, 95.2 kw and 50.9 kw after stabilization, respectively. And the variation pattern of oil temperature and heat transfer power with flow rate shown in Fig. 5(c) is basically consistent with that of Fig. 5(b). The heat transfer power corresponding to the above three working conditions are 134.5 kw, 89.5 kw and 52.1 kw respectively. Compared with the prototype, when the flow rates are 34.65 kg/s, 21.73 kg/s and 11.18 kg/s, the heat transfer power of the cooler with spiral flat tubes is increased by 73.8%, 62.2% and 43.8%, respectively, and that of the cooler with the adjusted bulkhead position is increased by 56.4%, 52.4% and 47.2%, respectively.

Comparing the results of the two optimization methods with the prototype one, it is found that the heat transfer power is substantially increased for different cooling medium flow rates. This phenomenon indicates that the proposed optimization approach of spiral twisted flat tube and bulkhead position is feasible and effective.

### 4.2 Analysis of numerical results

The above phenomena will be further analyzed in conjunction with velocity contour, pressure contour and temperature distribution in cooler tubes.

**Figure 6:**
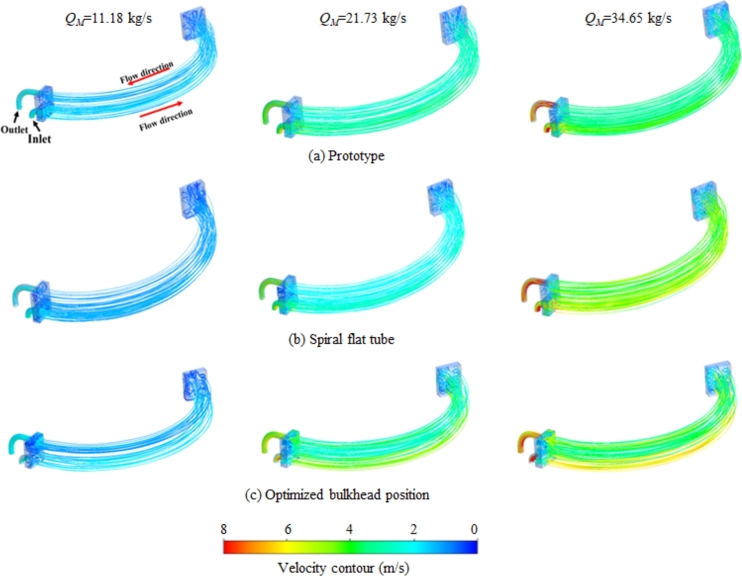
Velocity contours corresponding to 11.18 kg/s, 21.73 kg/s and 34.65 kg/s mass flow rates for coolers of (a) prototype, (b) spiral flat tube and (c) optimized bulkhead position.

Fig. 6(a) shows the velocity distribution of prototype for different mass flow rates. Cooling medium in the pipe back end to produce more intense turbulence and more vortexes, which reduces the fluid flow velocity and generates a large flow loss. The existence of flow dead zones in coolers is presented as a direction for structural optimization as well. Flow velocity distribution in the heat exchanger tube is relatively uniform, and the mass smaller flow rate makes the cooling medium flow velocity distribution more uniform. Moreover, velocity of the cooling medium in pipe's lower half is higher than that of the upper half. The velocity contours inside the spiral flat tube are shown in Fig. 6(b). Compared to the prototype, the medium flow velocity inside the spiral twisted flat tube is lower and the residence time is longer, which makes the heat transfer effect better. It is because that the spiral channel causes longitudinal rotation and secondary rotation, and this helical perturbation effect enhances the fluid disturbance degree, thins the boundary layer, and enhances the mixing of fluid, which improves the heat transfer coefficient of the fluid near pipe wall. When the bulkhead position is optimized, the difference in flow velocity in the upper part of heat exchanger tube creates more turbulence, which is beneficial for flow heat transfer, as shown in Fig. 6(c).

**Figure 7:**
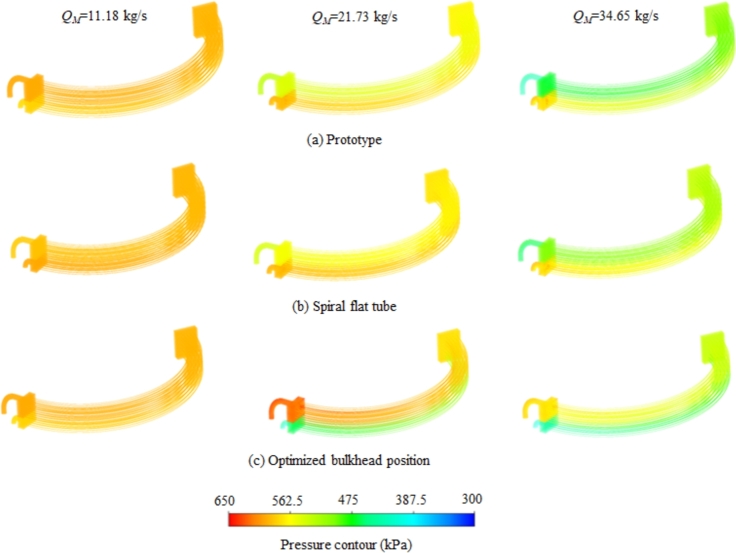
Pressure contours corresponding to 11.18 kg/s, 21.73 kg/s and 34.65 kg/s mass flow rates for coolers of (a) prototype, (b) spiral flat tube and (c) optimized bulkhead position.

The pressure contour of the medium in prototype cooler is demonstrated in Fig. 7(a). Since the cooling medium flow state is different in the upper and lower parts of cooler, a quite obvious pressure stratification is formed. When the mass flow rate is high, the lower inflow pressure is greater than the upper outflow pressure. And the phenomenon of cooling medium reflux will occur, which seriously affects the heat transfer performance, and it is also the reason for cooler's temperature stratification. For the spiral flat tube, this cooling medium reflux situation can not be reduced, as shown in Fig. 7(b), and the lower layer of the cooler pressure is still greater than the upper layer. When the position of the spacer is optimized, the phenomenon of medium reflux is effectively prevented. And more turbulence appeared in the upper heat exchanger tube, as shown in Fig. 7(c), which can improve the overall heat transfer effect.

**Figure 8:**
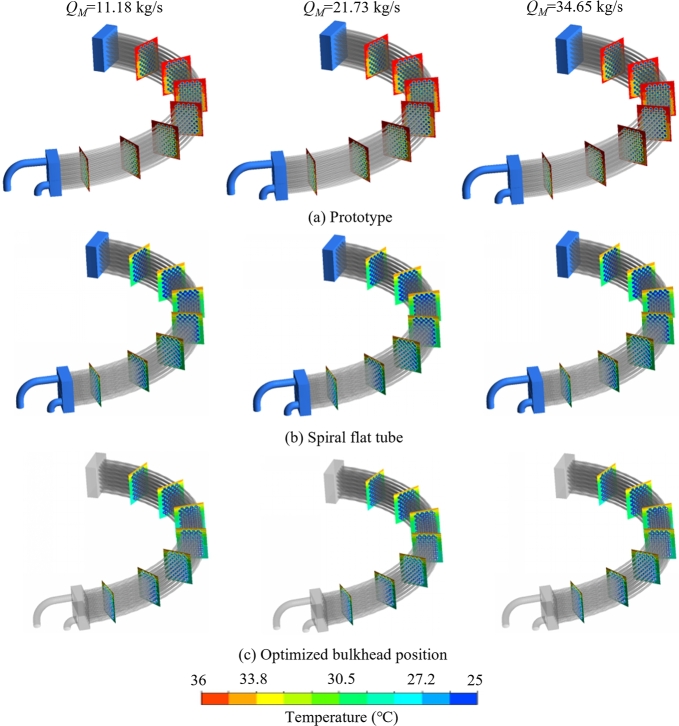
Radial plane temperature comparison contours corresponding to 11.18 kg/s, 21.73 kg/s and 34.65 kg/s mass flow rates for coolers of (a) prototype, (b) spiral flat tube and (c) optimized bulkhead position.

Fig. 8(a) demonstrates the temperature distribution in the radial planes $R_{1}$ to $R_{7}$. A large range of high temperature region exists in the upper region of the oil domain under different flow conditions, and this region is the dead zone of heat transfer, which needs to be optimized for its heat transfer performance. When the cooler is designed as the spiral flat tube, as shown in Fig. 8(b), the temperature stratification phenomenon only occurs in the upper half region of the heat exchanger, while the temperature distribution in the lower half region is more uniform, and compared with prototype, the heat transfer effect is greatly improved. As for the solution of optimizing the bulkhead position, more turbulence is generated in the cooling water by increasing fluid pressure in the upper cooler, which intensifies the convective heat transfer between the oil domain and cooling medium in tube's upper region. Overall the temperature field distribution range is reduced compared to the prototype cooler, which is given by Fig. 8(c).

**Figure 9:**
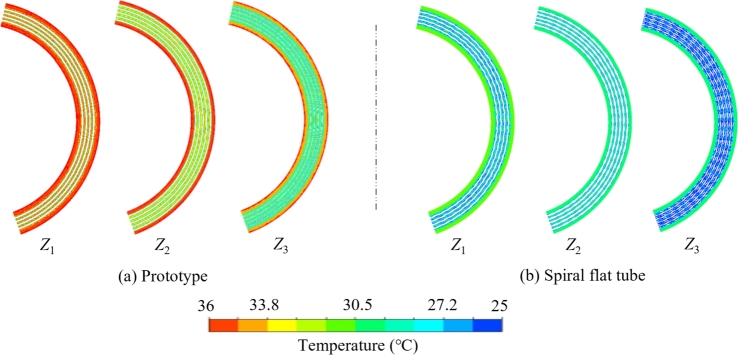
Axial plane temperature comparison contours corresponding to 34.65 kg/s mass flow rates for coolers of (a) prototype and (b) spiral flat tube.

Since both the heat transfer benefits from the optimization of the spiral flat tube and spacer position, only the axial cross-section of the spiral flat tube is chosen for comparison with prototype. The axial sections $Z_{1}$, $Z_{2}$ and $Z_{3}$ of the cooler are further analyzed by selecting the working condition of 34.65 kg/s, the result is shown in Fig. 9(a) and Fig. 9(b). Compared to the prototype cooler, the spiral flat tube cooler has a higher capacity for turbulent heat transfer. It produces a thinner thermal boundary layer, which enhances convective heat transfer between the fluid and pipe's wall. The $Z_{3}$ planar region has the widest range of low temperatures, with the lowest temperature close to the cooling medium's temperature. The overall heat transfer performance of the spiral flat tube cooler is significantly improved.

**Figure 10:**
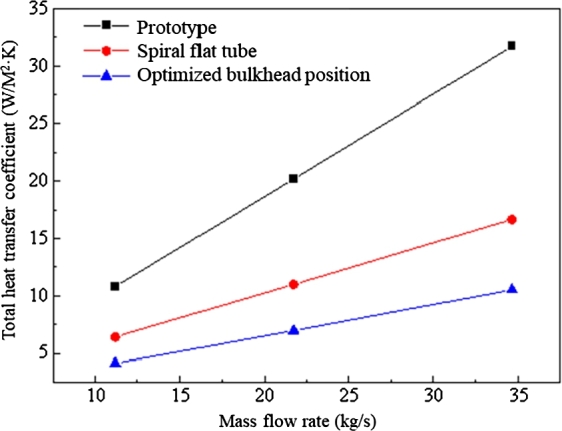
Variation of total heat transfer coefficient with mass flow rate for three design options.

The variation of total heat transfer coefficient with mass flow rate for the three coolers is shown in Fig. 10. The total heat transfer coefficients of the three coolers increase with mass flow rate and it is approximately linearly distributed. The heat dissipation effect of the spiral flat tube cooler and bulkhead position optimized cooler is much greater than that of the prototype cooler. Among them, the spiral flat tube has the optimum heat transfer performance.

Through the contour analysis and visual heat dissipation results of above numerical simulation, it can be concluded that the spiral flat tube and bulkhead optimization scheme can significantly improve the heat dissipation performance of cooler. For the spiral flat tube structure, this performance enhancement phenomenon is reflected on fluid dynamics and numerical heat transfer that, the bending structure of spiral flat tube makes the residence time of cooling water flow in tube longer. Meanwhile, the turbulence of the water flow is continuously formed in the twisted tube, and the perturbation has been greatly increased, which is capable of scouring the impurities and reducing the dirt thickness adhering to pipe wall, and this also improves the heat conduction efficiency. The cooler's heat transfer capability is also enhanced by moving the spacer position downwards. This is attributed that, the flow rate at copper pipe's inlet and the pressure at outlet are increased, an obvious pressure stratification is formed among the cooler's upper and lower parts, where the outflow pressure of upper layer is higher than the inflow pressure of lower layer. The higher pressure in upper layer increases the water's Reynolds number, where more turbulence is created in the upper heat exchanger pipes. Therefore, the pipes in upper layer can be effectively worked and the overall heat transfer effect can be improved.

## 5 Conclusion

In this paper, the feasible measures to enhance heat transfer are firstly derived by analyzing the heat transfer mechanism. On this basis, two improvement measures of spiral flat tube and bulkhead position optimization are proposed, a detailed numerical simulation study is carried out with pressure contour, temperature distribution and velocity field, and verification experiments are carried out on a 100 MW Francis turbine generator set. The main conclusions are as follows

(1) The spiral flat tube effectively reduces the risk of clogging of the heat exchanger tube, and improves the heat exchanger efficiency of the cooler, which enhances the reliability and stability of the condensing cooler for different media conditions.

(2) The heat transfer efficiency of the cooler is improved by 62.2% and 52.4% respectively after adopting the two optimized solutions of spiral flat tube and bulkhead position, when flow rate is 21.73 kg/s. And it effectively reduces the temperature field distribution of the shell side of the high temperature lubricating oil field, and balances the temperature stratification phenomenon of the upper and lower halves of the cooling medium inflow and outflow, which makes the overall temperature field distribution of the cooler more uniform.

## Ethical standards

The research meets all ethical guidelines, including adherence to the legal requirements of the study country.

## Funding

This study was supported by the Shaanxi Province Postdoctoral Research Project (Grant No. 2023BSHEDZZ257); the Natural Science Projects of Shaanxi Education Department (Grant No. 22JK0482) and the University-level Doctoral teacher Research Start-up Fund project (803-451121001).

## CRediT authorship contribution statement

**Yongfei Wang:** Resources, Methodology, Conceptualization. **Yinhui Cai:** Validation, Investigation. **Jian Zhang:** Software, Data curation. **Zhenyu Chen:** Writing – original draft, Software. **Chenhao Li:** Writing – review & editing, Supervision, Funding acquisition. **Weipeng Sun:** Resources, Formal analysis.

## Declaration of Competing Interest

The authors declare that they have no known competing financial interests or personal relationships that could have appeared to influence the work reported in this paper.

## Data Availability

Data will be made available on request.
